# Brain age prediction and early neurodegeneration detection using contrastive learning on brain biomechanics: a retrospective, multicentre study

**DOI:** 10.1016/j.ebiom.2025.105996

**Published:** 2025-10-31

**Authors:** Jakob Träuble, Lucy V. Hiscox, Curtis L. Johnson, Angelica Aviles-Rivero, Carola B. Schönlieb, Gabriele S. Kaminski Schierle

**Affiliations:** a Department of Chemical Engineering and Biotechnology, University of Cambridge, Cambridge, United Kingdom; b Cardiff University Brain Research Imaging Centre (CUBRIC), School of Psychology, Cardiff University, Cardiff, United Kingdom; c Department of Biomedical Engineering, University of Delaware, Newark, USA; d Yau Mathematical Sciences Center, Tsinghua University, China; e Department of Applied Mathematics and Theoretical Physics, University of Cambridge, Cambridge, United Kingdom

**Keywords:** Brain age prediction, Magnetic resonance elastography (MRE), Contrastive learning, Neurodegenerative diseases, Biomarkers

## Abstract

**Background:**

One of the main reasons why drugs for neurodegenerative diseases often fail is that treatment typically begins only after symptoms have appeared—by which point significant, and possibly irreversible, damage may have already occurred. Non-invasive imaging techniques, such as Magnetic Resonance Imaging (MRI), have previously been explored for presymptomatic diagnosis, but with limited success. More recently, Magnetic Resonance Elastography (MRE)—a technique capable of mapping the brain's biomechanical properties, including stiffness and damping ratio—has shown promise in detecting early changes. However, current studies have been limited by small sample sizes, and a lack of robust algorithms capable of accurately interpreting data under such constraints.

**Methods:**

We developed a self-supervised contrastive regression framework trained on 3D MRE-derived stiffness and damping ratio maps from 311 healthy individuals (aged 14–90) and evaluated its performance against structural 3D T1-weighted MRI. Brain age predictions were used to compute brain age gaps (BAGs), quantifying deviations from normative ageing trajectories. We applied the models to Alzheimer's disease (AD, n = 11) and mild cognitive impairment (MCI, n = 20) cohorts, and analysed whole-brain and region-specific predictions using occlusion-based saliency maps and subcortical segmentation.

**Findings:**

Self-supervised models using MRE achieved a mean absolute error (MAE) of 3.51 years in brain age prediction—significantly outperforming MRI (MAE: 4.79 years, p < 0.05) under matched conditions. The greater age sensitivity of MRE translated into improved differentiation of Alzheimer's disease (AD) and mild cognitive impairment (MCI) from healthy individuals. Stiffness was the dominant ageing biomarker in AD (BAG increase: +9.2 years, p < 0.05), whereas damping ratio revealed early MCI-related changes (BAG increase: +6.3 years, p < 0.05). Region-wise analysis identified the caudate (stiffness) and thalamus (damping ratio) as key markers for AD and MCI, respectively. Notably, some cognitively normal individuals exhibited biomechanical profiles resembling patients with MCI or AD, suggesting that these individuals may share some biomechanical characteristics with clinical populations.

**Interpretation:**

In our controlled experimental setting, MRE combined with contrastive learning provides a sensitive, non-invasive biomarker of brain ageing and neurodegeneration, outperforming MRI and differentiating disease stage–specific biomechanical signatures. Regional BAG profiling may have the potential to identify at-risk, cognitively normal individuals, which could facilitate timely intervention trials in the future, pending longitudinal validation.

**Funding:**

10.13039/501100005370Gates Cambridge Trust; Cambridge Centre for Data-Driven Discovery (Schmidt Sciences); 10.13039/100010269Wellcome Trust; 10.13039/100000002NIH (R01-AG058853, U01-NS112120); UK 10.13039/501100000266EPSRC; UK MRC; Alzheimer’s Research UK; 10.13039/100000864Michael J. Fox Foundation; Infinitus China Ltd.

**Research in context**

**Evidence before this study**

Magnetic Resonance Elastography (MRE) has emerged as a powerful and highly sensitive modality for quantifying brain tissue mechanics—such as stiffness and damping ratio—which are known to change with ageing and in neurodegenerative diseases. While previous studies have shown that these mechanical properties decline with age and are altered in AD, their predictive utility has been limited, largely due to small cohort sizes and coarse analytical approaches. To date, most MRE research has focused on whole-brain averages or simplistic linear trends, overlooking the potential of spatially resolved analysis and the integration of advanced machine learning models. In contrast to structural MRI, which has been extensively used for brain age prediction, the application of MRE in predictive modelling—especially using deep learning in the context of neurodegeneration—remains largely untapped. This represents a critical missed opportunity, given MRE's unique sensitivity to early and subtle tissue changes that precede structural atrophy.

**Added value of this study**

This study presents a powerful self-supervised contrastive regression framework tailored for small datasets, leveraging 3D MRE-derived maps of brain stiffness and damping ratio to predict brain age and uncover early neurodegenerative changes. Our model not only surpasses traditional MRI-based methods in age prediction accuracy but also uncovers distinct, spatially resolved mechanical ageing trajectories. Through brain age gap analysis and region-specific profiling, we identify the caudate and thalamus as critical age-sensitive hubs. Crucially, we demonstrate that damping ratio detects subtle early-stage changes in MCI, while stiffness reflects more advanced degeneration in AD. Most strikingly, our approach flags cognitively healthy individuals whose biomechanical signatures mirror those of disease cohorts—offering a transformative, non-invasive strategy for presymptomatic detection and intervention in neurodegenerative disease.

**Implications of all the available evidence**

MRE combined with contrastive learning offers a powerful, non-invasive tool for detecting early neurodegenerative changes and individual deviations from normative ageing. These findings suggest that biomechanical brain properties, particularly when analysed at high spatial resolution, could serve as robust biomarkers for risk stratification, early intervention planning, and longitudinal monitoring in clinical research on cognitive decline and dementia. Future work should explore integration with multimodal imaging and larger prospective studies.

## Introduction

Neurodegeneration and ageing are closely intertwined, yet few methods exist to non-invasively monitor their progression across the human lifespan. While different neurodegenerative diseases target specific brain regions, our understanding is largely derived from post-mortem analyses that capture end-stage pathology. Much less is known about how these diseases evolve over time and how ageing influences their onset and progression. Ageing itself is a multifaceted biological process that affects the brain at molecular, cellular, structural and functional levels.^1^, ^2^, ^3^ Over the past decades, neuroimaging techniques—aimed at providing unique perspectives on the key biological processes underlying neurodegeneration and ageing—have offered some insights into these disease- and age-related changes, revealing declines in grey matter volume, white matter integrity, and functional connectivity.^4^, ^5^, ^6^, ^7^

More recently, magnetic resonance elastography (MRE) has emerged as a promising method for characterising the biomechanical properties of brain tissue, capturing microstructural properties of neural tissue, which are relevant for brain ageing and neurodegeneration.^8^ Unlike conventional magnetic resonance imaging (MRI), which provides anatomical images and is typically used to measure brain volumes, MRE non-invasively characterises the brain's viscoelasticity by yielding quantitative maps of brain tissue shear stiffness, reflecting tissue composition, and damping ratio, which relates to cellular organisation.^9^ MRE involves a conventional MRI scanner but with an actuation system and specialised imaging pulse sequences to generate and track shear waves as they propagate through tissue, from which tissue mechanical properties are reconstructed via an inverse problem.^10^ Recent studies indicate that mechanical brain properties exceed traditional MRI measures in sensitivity to age-related changes.^8^^,^^11^^,^^12^ For instance, whole-brain stiffness has shown a sensitivity to ageing-related softening at a rate three times greater than volumetric atrophy rates observed with MRI.^13^ Furthermore, this sensitivity extends to neurodegenerative diseases such as Alzheimer's disease (AD), Parkinson's disease (PD), and frontotemporal dementia (FTD), where abnormal mechanical alterations in specific brain regions have been reported.^14^, ^15^, ^16^ There are some regional MRE studies that have shown that ageing effects are not uniform across the brain, with the frontal, temporal, and occipital lobes displaying distinct mechanical signatures of ageing, while deeper brain structures show differential responses depending on disease state.^17^^,^^18^ However, current MRE studies predominantly focus on whole-brain or region-wide averages, underutilising the detailed information available in biomechanical maps, which can be captured via nonlinear relationships at the voxel level. Unlocking this level of detail could provide a more refined understanding of localised mechanical changes associated with ageing and neurodegeneration, potentially enabling earlier detection when interventions may still be effective.

Brain age estimation—the prediction of chronological age from neuroimaging data—has gained prominence as a means to assess deviations from normative ageing trajectories.^19^ This technique has been predominantly applied to structural MRI modalities^20^ but has also been extended to other imaging techniques such as functional MRI (fMRI) and diffusion MRI.^21^ In the field of brain age estimation, different modelling approaches have been used, ranging from traditional statistical kernel methods such as Gaussian processes^22^ to deep learning models like convolutional neural networks (CNNs),^23^ and more recently, advanced self-supervised learning approaches.^24^ By leveraging a relatively large dataset of healthy individuals to establish a normative ageing trajectory, these models effectively mitigate class imbalance issues often found in classification tasks, where disease cohorts are typically underrepresented. Recently, Claros-Olivares et al.^25^ introduced MRE-based features for brain age prediction using CNNs, demonstrating the utility of mechanical biomarkers. However, progress has been limited by small sample sizes and a lack of robust algorithms capable of handling such data.

In this study, we present a new framework that integrates MRE-derived stiffness and damping ratio metrics to enhance brain age prediction and detect pathological ageing in neurodegenerative disease. In contrast to standard supervised convolutional networks that learn directly from individual image–label pairs, contrastive learning leverages relationships between pairs of images, enabling many more training examples to be generated from the same dataset — an advantage in small-sample settings such as ours. Furthermore, by incorporating adaptive neighbourhoods, the framework can guide representation learning from broad inter-subject differences toward finer-grained distinctions as training progresses. Our MRE-based approach shows a greater sensitivity in detecting age-related changes compared to conventional MRI-based models. Specifically, we find that stiffness helps capture late-stage neurodegenerative alterations in AD, while damping ratio is more sensitive to early changes in MCI. We further identify the caudate and thalamus as highly age-sensitive structures, with predominant thalamic involvement in AD and early hippocampal vulnerability in MCI.

Crucially, our framework can identify cognitively healthy individuals whose biomechanical ageing profiles resemble those of patients with MCI or AD. While longitudinal studies are needed for confirmation, this offers a promising avenue for developing a non-invasive method for early disease screening and personalised risk stratification.

## Methods

### Study design and participants

**Fig. 1:**
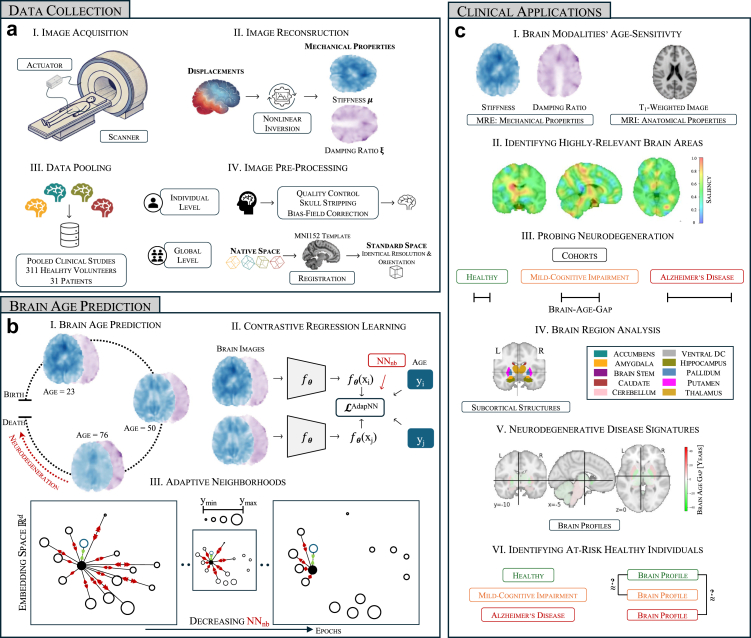
**Study workflow overview**. (a) Data collection and preprocessing: Image acquisition via MRE, reconstruction using nonlinear inversion to extract mechanical properties, data pooling from multiple clinical studies, and preprocessing steps such as skull stripping, bias-field correction, and registration to the MNI152 template. (b) Brain age prediction framework: A self-supervised contrastive regression model leveraging adaptive neighbourhood selection to enhance age-related feature learning. (c) Clinical applications: Predicting brain age trajectories during healthy ageing to compare age-sensitivity of brain modalities, visualising most relevant brain areas, probing neurodegeneration using modelled normative ageing trajectories, assessing regional differences across ageing and disease, and identifying at risk healthy individuals showing similar brain profiles to disease signatures.

This retrospective, multi-centre study pooled MRE datasets from five healthy-volunteer studies (n = 311; 14–90 years)^11^^,^^16^^,^^26^, ^27^, ^28^, ^29^ and two patient cohorts (mild cognitive impairment [MCI], n = 20; Alzheimer's disease [AD], n = 11).^16^^,^^26^ The pooled dataset comprises structural MRI and MRE data, collected under highly similar acquisition protocols. For each participant, mechanical properties are extracted by applying nonlinear inversion techniques^30^ to brain tissue displacement data collected with MRE, resulting in quantitative maps of stiffness μ and damping ratio ξ.^31^^,^^32^ Subsequent preprocessing—including skull stripping and bias-field correction using FreeSurfer,^33^ and registration to the MNI152 template using ANTs^34^—ensures that these maps are spatially standardised (Fig. 1a). These standardised maps serve as inputs for predictive modelling. Full demographic tables, inclusion criteria and detailed pre-processing workflows are provided in Supplementary Material (pp 2 and 3).

### Algorithm development and procedures

We have developed a two-stage contrastive regression framework to predict age from brain images. In the first stage, the encoder is trained with an age-aware contrastive objective that arranges subjects in a latent space so that similar ages are closer together and dissimilar ages are farther apart. An adaptive neighbourhoods mechanism progressively narrows the set of repelled neighbours during training, shifting the focus from coarse to fine age distinctions. Once the encoder is trained, it is frozen, and a ridge regression model is fitted on the latent representations of the training set to predict age.

Central to our brain age prediction framework is the adaptive neighbourhood approach—a contrastive learning method tailored for regression tasks under non-uniform distributions in low-data regimes, which we have recently developed for stiffness maps.^35^ In this work, we have extended this self-supervised framework to utilise additional neuroimaging data, such as damping ratio also from MRE and MRI-derived anatomical images for comparison (Supplementary Material pp 4 and 5). Furthermore, we expand this framework to facilitate the integration of segmentation-based subcortical regions. Here, our contrastive learning method dynamically adjusts sample neighbourhoods to emphasise age-relevant differences. The adaptive contrastive loss for a sample x_i_ is defined as:

$$L_{\text{AdapNN}}=-\sum_{i}\sum_{k\neq i}\frac{w_{i,k}}{\sum_{t}w_{i,t}}\log\left(\frac{\exp\left(s_{i,k}\right)}{\sum_{x_{t}\in \text{NN}\left(x_{i};\text{epoch}\right)}\exp\left(s_{i,t}\left(1-w_{i,t}\right)\right)}\right)$$

where w_i,k_ = K (y_i_ - y_k_) measures the similarity in age between samples x_i_ and x_k_ via a Gaussian kernel function K (·), and s_i,k_ = sim (f (x_i_), f (x_k_)) measures the similarity of their feature embeddings.

Thus, both w_i,k_ (age-kernel weight) and s_i,k_ (embedding similarity) are computed at the subject level. The dynamically adjusted set NN (x_i_; epoch) ensures that the loss function focuses on the most relevant comparisons at each training stage. This allows the model to capture localised ageing patterns and to generalise across the heterogenous dataset (Fig. 1b).

We benchmarked our proposed contrastive framework against two baseline models representing the dominant approaches in the field: (1) a kernel-based method using principal-component analysis^36^ followed by Gaussian-process regression,^37^ (2) a fully supervised deep learning approach using a 3-D ResNet-18 convolutional neural network.^38^ All models were trained using an 80:20 train-test split. Model performance was assessed using the mean absolute error (MAE) on the held-out test set, averaging the model's brain age predictions across ten random seeds. For MRE models, stiffness (μ) and damping-ratio (ξ) volumes were supplied as separate input channels, enabling each convolutional kernel to learn joint spatial patterns across the two modalities. Detailed hyperparameter tuning strategies and model configurations are described in the Supplementary Material pp 5–7. To visualise spatial drivers of age prediction, occlusion-based saliency maps^39^ were generated on the independent test set. For each subject, 7 × 7 × 7 voxel regions were masked and the change in prediction error was computed (ΔMAE). Maps were generated across five age bins and normalised to allow group-level visualisation. Regions with the largest error increase upon occlusion were considered most influential for the model. For both whole-brain and regional analyses, MAE values were calculated on the held-out test set of healthy participants to assess raw prediction performance. We quantified deviations from normative ageing trajectories using the brain age gap (BAG), calculated as the difference between predicted and chronological age. For disease cohorts, models were retrained using only healthy individuals (excluding matched controls), and BAGs were bias-corrected using a Theil-Sen regressor^40^, ^41^, ^42^, ^43^ fitted on the control group. BAGs were computed for the whole brain and ten subcortical regions. By integrating voxel-wise MRE-based measurements with advanced adaptive learning strategies, our approach (I) compares MRE to MRI in age sensitivity (II) pinpoints brain areas highly relevant for predictions (III) evaluates stiffness and damping ratio in neurodegeneration (IV) analyses localised ageing effects (V) establishes neurodegenerative disease signatures in deep brain structures and (VI) highlights healthy individuals with brain age profiles that resemble those of clinical cohorts, as illustrated in Fig. 1c.

### Ethics

This study is a retrospective, secondary analysis of fully de-identified data pooled from previously published studies. All datasets included in this analysis were collected in accordance with ethical standards, under protocols approved by the respective local institutional review boards of the original studies.^11^^,^^16^^,^^26^, ^27^, ^28^, ^29^ In each of these primary studies, all participants provided written informed consent for their participation, which included consent for the sharing and re-analysis of their de-identified data for future research.

### Statistics

We used the Shapiro-Wilk^44^ test to assess normality. To compare the performance between different model configurations (e.g., MRI vs. MRE), we used either paired t-tests^45^ or the non-parametric Wilcoxon signed-rank^46^ test depending on whether the data were normally distributed or not. For these paired tests, the dependent variable was the Mean Absolute Error (MAE) from each of the 10 model training runs. The 95% confidence intervals for the mean absolute error were calculated using the t-distribution from the results of the 10 model training runs. To compare the Brain Age Gap (BAG) between clinical cohorts, we used independent t-tests^45^ or the non-parametric Mann–Whitney U test.^47^ For these group comparisons, the dependent variable was the calculated BAG, and the independent group variable was the participant's clinical diagnosis (Healthy, MCI, or AD), with each disease cohort compared only to its own matched healthy control group using independent samples. All tests were two-sided with significance set at p < 0.05. All analyses were performed in Python using SciPy.

### Role of funders

The funders of the study had no role in study design, data collection, data analysis, data interpretation, or writing of the report.

## Results

### MRE outperforms MRI in brain age estimation, with brain stiffness as the dominant ageing biomarker

**Fig. 2:**
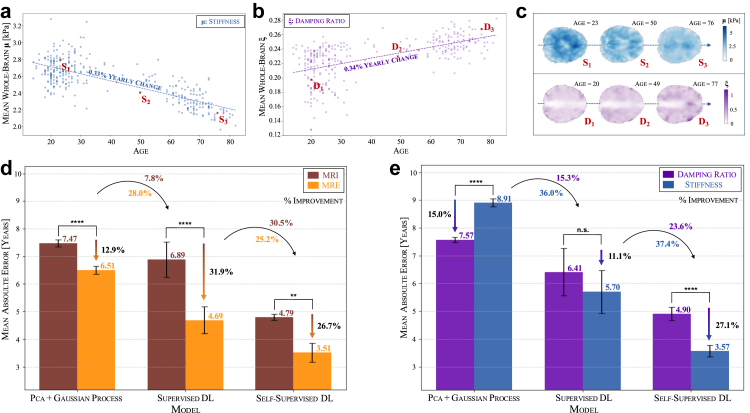
**Whole-brain biomechanical age trends reveal superior performance of voxel-wise MRE-based models over MRI in brain age prediction**. (a) Whole-brain average stiffness μ decreases with age, while (b) damping ratio ξ increases, reflecting distinct ageing trajectories. (c) Representative mechanical property maps illustrate brain softening and increasing viscoelasticity across different ages. (d) Comparison of mechanical (MRE) against anatomical properties (MRI) using three distinct modelling approaches shows improved performance of MRE and that self-supervised deep learning achieves the lowest mean absolute error (MAE) for brain age prediction, outperforming PCA + Gaussian Processes and supervised deep learning. (e) Comparison of unimodal models (stiffness-only and damping ratio-only) highlights stiffness as the dominant mechanical biomarker of ageing. Statistical significance between modalities was assessed using paired t-tests or Wilcoxon signed-rank tests. n.s. (not significant) p ≥ 0.05; ∗∗p < 0.01; ∗∗∗∗p < 0.0001. N = 10.

To validate that our model inputs reflect biologically meaningful ageing trends, we first examined whole-brain average stiffness μ and damping ratio ξ across age. Global stiffness declines at a rate of −0.33% per year, highlighting progressive brain softening with ageing (Fig. 2a)–consistent with trends observed in the field.^13^^,^^17^^,^^18^ In contrast, the damping ratio increases at a rate of 0.34% per year, indicating more viscous or fluid-like tissue behaviour over time with higher energy dissipation (Fig. 2b). Representative mechanical brain maps at three different ages (Fig. 2c) illustrate these effects, showing a clear reduction in stiffness alongside the increase in damping properties. The strong association of both mechanical properties with age supports their suitability for brain age prediction. Building on these whole-brain trends, we utilise a voxel-wise approach to enhance spatial resolution, allowing models to capture localised ageing patterns across the brain.

We compare three distinct modelling approaches for brain age prediction (Fig. 2d). The results demonstrate a improvement from established to more recent machine learning approaches, highlighting the impact of advanced modelling techniques on brain age prediction accuracy. While PCA with Gaussian processes achieves a mean absolute error (MAE) of 7.47 years (95% CI: 7.38–7.57) using MRI, deep learning improves prediction accuracy, with supervised deep learning reducing the MAE to 6.89 years (95% CI: 6.41–7.37) and self-supervised learning further lowering it to 4.79 years (95% CI: 4.71–4.87). This trend is even more pronounced when incorporating MRE-based properties—namely, stiffness and damping ratio—where the MAE decreases from 6.51 years (95% CI: 6.40–6.61) with PCA + GPs to 4.69 years (95% CI: 4.32–5.06) with supervised deep learning, and further to 3.51 years (95% CI: 3.26–3.77) with self-supervised learning. Notably, in our dataset and experimental setup, self-supervised learning improved MRE-based predictions by 25.2% compared to the supervised baseline. This gain reflects the advantage of advanced representation learning under conditions where model capacity is matched to dataset size (ResNet-18 for the supervised baseline) and training protocols are harmonised across modalities. Across all three model classes, the MAE is consistently lower when using mechanical brain properties derived from MRE compared to traditional T1-weighted MRI scans. For PCA + GPs, the difference between MRI and MRE is relatively small, with only a 12.9% reduction in MAE (t (9) = 15.48, p < 0.001). However, for deep learning models, the advantage of MRE becomes much more pronounced, with supervised deep learning showing a 31.9% reduction in MAE (t (9) = 11.08, p < 0.001) and self-supervised learning achieving a similar 26.7% reduction (W = 0, p < 0.002). MRE-based models showed lower MAEs than MRI-based models across all three methodological families, indicating higher age sensitivity of mechanical properties under matched conditions.

We now assess stiffness and damping ratio individually (Fig. 2e). While both mechanical properties capture ageing-related changes, their effectiveness depends on the modelling approach. For kernel methods, damping ratio achieves a lower MAE (7.57 years; 95% CI: 7.50–7.64) compared to stiffness (8.91 years; 95% CI: 8.80–9.02), showing a 15.0% improvement (t (9) = 46.20, p < 0.001). This may be due to the more diffuse spatial distribution of damping ratio, which could align better with the global nature of kernel-based representations. However, with more advanced models, stiffness emerges as the more informative feature for accurately modelling the ageing trajectory. Supervised deep learning reduces the MAE to 6.41 years (95% CI: 5.77–7.06) with damping ratio and further to 5.70 years (95% CI: 5.11–6.28) with stiffness, a 11.1% improvement, however this difference was not statistically significant (t (9) = −1.69, p = 0.13). With self-supervised learning, stiffness-based predictions achieve an MAE of 3.57 years (95% CI: 3.42–3.72), a substantial 27.1% improvement over damping ratio (t (9) = −10.84, p < 0.001), reinforcing stiffness as the more informative feature. This reflects the ability of deep learning models to capture more focal, spatially specific ageing patterns observed in stiffness maps. In general, combining stiffness and damping ratio—as shown in Fig. 2d—tend to yield better accuracy than using either property alone. For instance, in our best-performing self-supervised model, the combined MRE model significantly outperforms the damping ratio-only model (W = 0.0, p = 0.002). Remarkably, for self-supervised learning, stiffness alone (3.57 years; CI: 3.42–3.72) performs similarly to the combined MRE model (3.51 years; CI: 3.26–3.77; W = 16.0, p = 0.28), implying that, with sufficiently robust representation learning, stiffness alone captures most relevant age-related information.

We further investigate the spatial effects of ageing (Supplementary Material p 8) by normalising each image independently, removing the global trends of brain softening and increasing viscoelasticity observed in Fig. 2a–c. Under these spatially normalised conditions, MRE-based predictions remain highly effective, confirming that stiffness and damping ratio capture meaningful ageing signals beyond global trends. This demonstrates that mechanical properties encode additional localised ageing effects that persist independently of whole-brain trends, further highlighting the unique sensitivity of our MRE-derived biomarkers.

To explore the interplay between the modalities, we have also evaluated models trained on a combination of MRI and MRE data, which shows improved performance over MRI alone but yields results comparable to MRE-only models (Supplementary Material p 10), suggesting that MRE already captures most of the age-relevant information.

### Occlusion-based saliency maps reveal the importance of deep brain structures as markers for late life stages

**Fig. 3:**
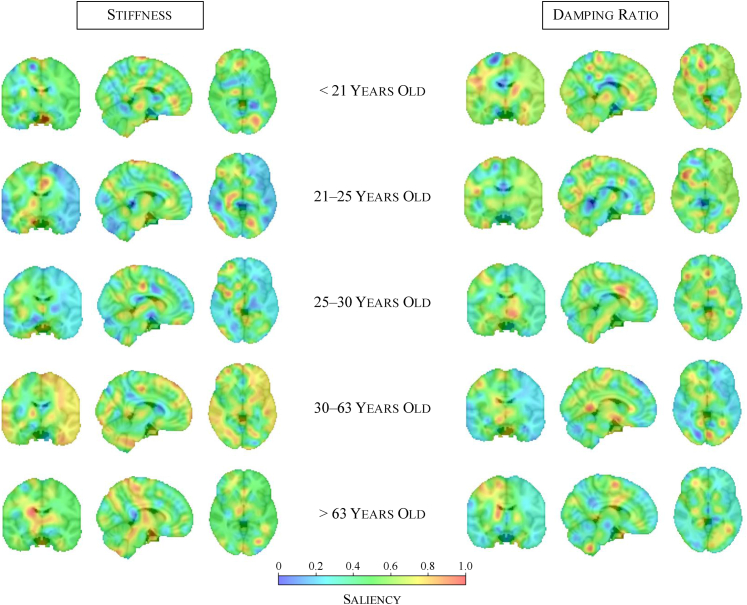
**Occlusion-based saliency maps highlight age-specific contributions of brain regions to brain age prediction by identifying which regions most strongly influence the model's output**. Each row represents a different quantile-based age bin, illustrating the spatial distribution of important features for stiffness-based models (left) and damping ratio-based models (right). Stiffness analysis reveals more focalised effects, with increasing cortical contributions in midlife and subcortical focus in older adults. Damping ratio exhibits a more diffuse distribution across the brain, suggesting broader viscoelastic ageing effects.

To interpret and gain insights into the brain age prediction models, we apply occlusion-based saliency maps (see Methods) to examine which spatial features contribute most to the model predictions (Fig. 3).

The saliency maps reveal how the model focus shifts across the brain with age, reflecting changes in the spatial importance of mechanical properties for brain age prediction. At the same time, they highlight distinct patterns across biomechanical modalities. For the stiffness-based model, saliency appears more fragmented in younger age groups, with multiple small regions of high importance primarily in deeper brain structures, while some high-saliency areas also emerge near the cortical surface. In contrast, for midlife individuals (30–63 years), saliency becomes more spatially coherent, with a smoother distribution that is more prominently focused on cortical regions. This shows that stiffness alterations in cortical areas may be stronger indicators of ageing during this period. In older age groups, the saliency distribution remains smooth rather than fragmented, in contrast to younger individuals. However, during older age, the model shifts its focus to subcortical structures, particularly the thalamus. Overall, deep grey matter structures, including the thalamus and putamen, consistently stand out on saliency maps across age groups, reinforcing their relevance in brain ageing.^18^^,^^48^ While the caudate, thalamus and putamen have previously been identified as the regions showing the most stiffness-related changes from childhood to adulthood,^49^ our findings extend the importance of the thalamus in stiffness-related ageing to later life stages.

For damping ratio-based predictions, the saliency maps reveal a more spatially diffuse pattern compared to stiffness, suggesting that damping ratio captures broader mechanical ageing effects. In younger age groups, high-saliency regions are distributed across the brain, spanning both deep grey matter structures and more cortical regions. However, in midlife and older age groups, while the overall saliency distribution remains diffuse, contributions from cortical regions diminish, and the model increasingly focuses on deeper brain structures. Among these, the caudate continues to show strong contributions across all age groups. Our findings show that the importance of cortical grey matter does not extrapolate to later life, whereas the caudate remains a key region throughout ageing. This extends previous studies^49^ that reported significant decreases in damping ratio within both the caudate and cortical grey matter during development from childhood to adulthood (ages 5–35 years), highlighting that while cortical effects are transient, the caudate's relevance persists beyond early-life changes. The overall more diffuse saliency pattern suggests that viscoelastic ageing effects, as captured by damping ratio, are not tightly confined to specific structures but rather reflect broader mechanical alterations that are spatially widespread, yet still informative at a local level.

These findings show that stiffness and damping ratio provide complementary information in brain age prediction, consistent with the improved performance observed in multi-modal models. While stiffness-based predictions exhibit a shifting spatial focus—initially more widespread in younger individuals, becoming more cortical in midlife, and then predominantly subcortical in older age—damping ratio-based predictions follow a more diffuse pattern, suggesting a more globally distributed role of viscoelastic changes in ageing. The prominence of deep grey matter structures, particularly the thalamus, in both modalities highlight their fundamental role in brain ageing, while the modality-specific spatial differences further support the idea that stiffness and damping ratio capture distinct but interrelated aspects of neurobiological ageing.

### Superior age sensitivity of MRE translates to improved detection of disease pathology of patients with AD and MCI

**Fig. 4:**
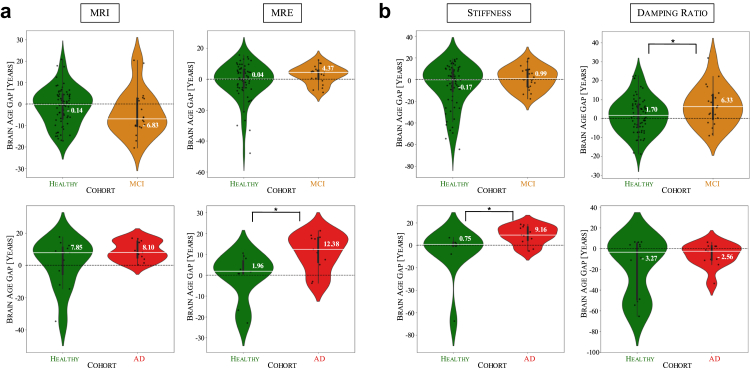
**Brain age gap (BAG) analysis highlights MRE's sensitivity to detecting neurodegeneration-related defects in patients with MCI and AD**. (a) MRI-based predictions show minimal differentiation in brain age gap (BAG) between healthy and diseased groups, while MRE-based models, using stiffness and damping ratio, capture an increase in the brain age gap in MCI and AD. (b) Stiffness is more sensitive to AD-related changes, while damping ratio captures early-stage neurodegeneration-related defects in MCI. Median values are annotated. Statistical significance between patient and control groups was assessed using independent t-tests or Mann–Whitney U tests. ∗p < 0.05. N_MCI_ = 20; N_MCI control_ = 68; N_AD_ = 11; N_AD control_ = 12.

We apply the self-supervised brain age models to cohorts of MCI and AD, and examine the brain age gap (BAG), i.e., the difference between predicted and chronological age, to assess whether the superior age sensitivity of MRE over MRI, observed earlier in healthy samples, translates into improved disease detection (Fig. 4a). BAGs are computed relative to normative trajectories learnt from healthy individuals, and predictions are evaluated on independent study-specific healthy control and patient groups. Applying the MRI-based models to the MCI cohort, the predicted age distribution shows a similar but slightly decreased profile to the healthy samples (mean of BAG: −4.00 years, median of BAG: −6.83 years), and this difference is not statistically significant (t (86) = 1.22, p = 0.225). In contrast, MRE-based predictions show an increase in the brain age gap, with the median shifting from 0.04 years in healthy samples to 4.37 years in MCI samples, thus, observing an increase in the brain age gap. However, this trend does not reach statistical significance (U = 546.0, p = 0.184). For the AD cohort, MRI-based predictions again show similar median values between healthy volunteers (7.85 years)—reflecting study-specific distributional differences relative to the normative trajectories—and patients (8.10 years), with no detectable statistical significance between cohorts (U = 50.0, p = 0.340). In contrast, MRE-based predictions reveal a statistically significant increase in BAG, with the median rising from 1.96 years in healthy samples to 12.38 years in patients with AD (U = 22.0, p = 0.022). These findings suggest that the greater age sensitivity of MRE over MRI observed in healthy ageing encodes meaningful information for detecting early neurodegeneration, as it translates to superior differentiation in disease cohorts.

To further dissect the individual contributions of stiffness and damping ratio to these effects, we examine their performance using stiffness-only and damping ratio-only models (Fig. 4b). In the MCI cohort, stiffness exhibits only a minimal increase in median BAG compared to normal controls (from −0.17 to 0.99 years), a non-significant difference (U = 614.0, p = 0.514), while damping ratio shows a statistically significant increase (from 1.70 to 6.33 years; t (86) = −2.95, p = 0.044), indicating BAG elevation in MCI was most strongly associated with changes in damping ratio. Interestingly, the combined MRE model does not outperform damping ratio alone, implying that stiffness neither strongly reinforces nor counteracts the observed trend. In contrast, in AD, stiffness-based predictions show a significant increase in BAG (from 0.75 to 9.16 years; U = 20.0, p = 0.015), while damping ratio exhibits only a minor, non-significant shift in median values (U = 67.0, p = 0.975). This suggests that BAG elevation in AD is most strongly associated with changes in stiffness, while damping ratio provides additional complementary information that enhances the MRE-based model's performance. Notably, unlike in MCI, the combined MRE model significantly outperforms damping ratio alone in AD (W = 0.0, p = 0.002), indicating that the integration of stiffness and damping ratio provides a more comprehensive characterisation of disease-related brain ageing.

### Regional brain age prediction reveals caudate and thalamus as key age-sensitive structures

**Fig. 5:**
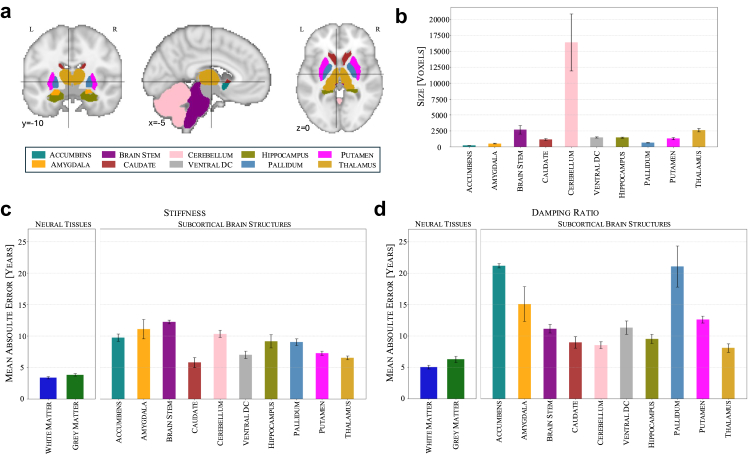
**Subcortical structures, white matter and grey matter display distinct patterns in stiffness and damping ratio, and caudate and thalamus are key age-sensitive structures as revealed by brain age prediction errors**. (a) Visualisation of ten subcortical structures used in the analysis. (b) Variability in anatomical volumes across regions. (c) Stiffness-based brain age prediction across subcortical regions shows caudate exhibits the lowest mean absolute error (MAE). (d) Damping ratio-based brain age prediction across subcortical regions shows thalamus displays the lowest MAE.

We extend the brain age modelling framework to individual brain regions, focussing on deep brain regions, which were identified as highly relevant in the whole-brain models through occlusion analysis earlier, as well as white matter (WM) and grey matter (GM), to investigate regional contributions to brain ageing. Fig. 5a visualises the segmentation of the ten subcortical structures, illustrating the spatial distribution of the selected regions. The corresponding region sizes, shown in Fig. 5b, highlight the substantial variability in anatomical volume across structures, ranging from large regions such as the cerebellum (mean: 16,408 voxels) and thalamus (2603 voxels) to smaller structures such as the nucleus accumbens (200 voxels) and the pallidum (633 voxels).

Using the self-supervised learning framework, we assess region-wise brain age prediction separately for stiffness and damping ratio by training models using all voxels within segmentation-based masks for each subcortical structure (Fig. 5c and d). For both stiffness and damping ratio, GM and WM exhibit lower MAEs than any individual subcortical structure, likely due to their larger volume, which provides a more stable signal for model training. GM consistently shows higher MAEs than WM, with stiffness-based predictions yielding MAEs of 3.83 years for GM and 3.38 years for WM, while damping ratio-based predictions result in 6.27 years for GM and 5.03 years for WM. This finding may reflect differences in the underlying tissue composition and ageing processes, as age-related changes in WM, such as demyelination and axonal degradation, are often more pronounced compared to GM. Furthermore, prior studies^50^ have shown that brain stiffness is correlated with myelin content, suggesting that changes in myelination could influence the predictive performance of stiffness-based models.

Among subcortical structures, the caudate yields the lowest mean absolute error (MAE) for stiffness-based predictions (5.78 years), while the thalamus achieves the lowest for damping ratio (8.07 years). These MAEs are expected to be higher than for whole-brain models because each regional model is trained on substantially fewer voxels, which limits the amount of age-relevant information available for prediction. Notably, both structures perform well across modalities, with the thalamus also showing low stiffness-based error (6.54 years) and the caudate performing reliably in damping ratio-based predictions (8.97 years). The hippocampus and ventral diencephalon (Ventral DC), of particular interest due to their known roles in the early development of neurodegenerative disease, exhibit moderate prediction accuracy (MAEs: 9.15 and 7.01 years for stiffness; 9.51 and 11.31 years for damping ratio, respectively). This region-wise analysis sheds further light on the contribution of individual deep brain structures to brain ageing by evaluating their predictive capacity in isolation—unlike saliency-based maps, which reflect more distributed patterns. By disentangling regional contributions, it highlights the caudate and thalamus as particularly informative structures for capturing age-related brain changes.

### Detecting regional BAG profiles in healthy individuals that resemble disease-related patterns and identifying thalamus and hippocampus as early markers of AD and MCI, respectively

**Fig. 6:**
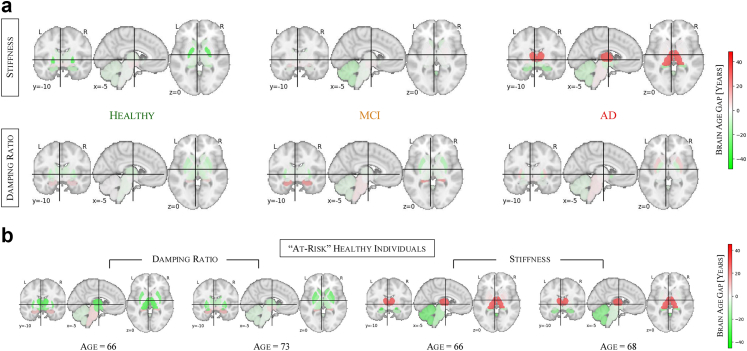
**Brain age profiling reveals distinct biomechanical alterations in MCI and AD, illustrating regional BAG patterns in some healthy individuals that resemble patient profiles**. (a) Subcortical brain age gaps (BAGs) show distinct regional patterns, highlighting the spatial heterogeneity in MCI and AD, with stiffness capturing AD-related changes, particularly a pronounced BAG elevation in the thalamus, and damping ratio highlighting early alterations in MCI, most notably in the hippocampus. (b) Individual examples of healthy subjects with brain age profiles similar to those observed in MCI or AD.

Having extended the brain age modelling framework to individual brain regions in healthy individuals, we now extend this regional analysis to disease cohorts to examine how neurodegenerative conditions affect mechanical brain ageing (Fig. 6a). We compute brain age gaps (BAGs) for each region in MCI and AD cohorts, allowing us to construct subcortical brain age gap profiles for each group. To create a representative cohort profile, we average the individual brain profiles within each cohort.

In stiffness-based brain age profiles, healthy individuals show predominantly neutral to slightly negative BAGs across brain regions, suggesting that their predicted brain ages align closely with or are slightly younger than their chronological age. In the MCI cohort, the regional brain age profile is varied, with most regions showing only minor deviations from chronological age, though a moderately elevated BAG is observed in the amygdala (8.19 years). A distinct shift in this pattern emerges in the AD cohort, which is characterised by a markedly pronounced BAG elevation in the thalamus (47.31 years). More subtle elevations are also present in the pallidum (3.59 years) and brain stem (2.88 years). The progression from healthy to AD is reflected in these brain age profiles, with increasing regional deviations marking the transition from normal ageing to neurodegeneration. These findings mirror the whole-brain results, where stiffness shows the strongest association with BAG elevation in AD. In damping ratio-based brain age profiles, healthy individuals similarly exhibit neutral to negative BAGs across most regions. However, in the MCI cohort, the hippocampus shows the strongest elevation (26.46 years), followed by the brain stem (5.24 years), while the ventral diencephalon exhibits only a slight increase (0.60 years), suggesting early-stage neurodegenerative changes. Unlike stiffness-based profiles, damping ratio-based predictions do not show a progressive increase in BAGs from MCI to AD. Instead, the AD cohort exhibits a brain profile similar to MCI, with the putamen showing the highest BAG elevation (11.16 years), while the brain stem (5.10 years) remains elevated but without further progression, suggesting that viscoelastic changes are more prominently associated with early neurodegenerative processes than with later-stage disease progression.

Overall, these subcortical brain profiles provide regional insights into neurodegeneration and further support the differential contributions of stiffness and damping ratio to disease detection—stiffness being more sensitive to AD-related changes and damping ratio capturing early-stage alterations in MCI.

Beyond group-level analyses, these regional BAG profiles allow us to examine individual healthy participants and assess whether their brain age patterns resemble those observed in MCI or AD cohorts. Fig. 6b shows four such examples, where the first two healthy individuals are damping ratio-based profiles closely resembling the MCI cohort profile, while the latter two healthy individuals display stiffness-based profiles similar to the AD cohort. While the underlying causes remain to be determined, the resemblance between the BAG profiles of these healthy individuals and the clinical cohorts is noteworthy.

## Discussion

Within our controlled design, MRE-based models consistently showed lower MAEs than MRI-based models in brain age prediction, especially when using modern self-supervised learning techniques, such as the contrastive learning method developed here, which is particularly well suited for small sample sizes. This superior sensitivity to age-related change suggests that MRE can detect subtler brain ageing effects than structural MRI. Structural MRI predominantly captures age-related macrostructural changes, such as volumetric atrophy, cortical thinning, and ventricular enlargement—relatively coarse markers that tend to emerge in later stages of ageing.^4^^,^^51^^,^^52^ In contrast, our findings align with physiological evidence that biomechanical brain properties are sensitive to earlier microstructural alterations that may arise closer to the underlying molecular and cellular changes of ageing, and which often remain undetected by conventional MRI.^8^^,^^9^^,^^53^^,^^54^ MRE effectively captures these subtler alterations, including changes in neuronal density, myelination, and extracellular matrix organisation—hallmarks of the ageing process.^9^^,^^55^, ^56^, ^57^, ^58^ Although one recent study^25^ applied deep learning to MRE-derived features, most prior MRE studies remain restricted to linear, whole-brain, or region-wide averaging methods, all of which are further limited by small datasets.^14^^,^^17^^,^^18^^,^^59^ Our voxel-wise approach moves beyond these assumptions, capturing non-linear biomechanical dependencies at high spatial resolution. Leveraging a contrastive regression framework tailored to non-uniform, low-data regimes,^60^ our method reveals finer-grained mechanical ageing patterns, outperforming both kernel-based and supervised deep learning models.

Our results reinforce the notion that stiffness is the dominant mechanical biomarker of brain ageing, but crucially, it is the application of our algorithmic framework that allows this property to be fully leveraged. The predominance of stiffness over damping ratio reflect their distinct biophysical underpinnings: stiffness is more directly influenced by tissue composition, whereas damping ratio is thought to relate to cellular organisation.^9^ This finding thus emphasises the greater relevance of tissue composition in capturing age-related brain changes, supporting prior observations that stiffness declines strongly with age and disease—while damping ratio shows less pronounced changes.^8^

Our occlusion-based saliency analysis pinpoints regions with arbitrary shapes and sizes of most interest to brain age models. Notably, the saliency maps reveal a shift in the most predictive brain regions across different age groups, supporting previous findings that mechanical ageing patterns evolve throughout the lifespan.^12^^,^^54^^,^^59^ Stiffness-based brain age models shift focus from cortical to subcortical regions, increasingly emphasising the thalamus and caudate in older adults, extending their known role in mechanical ageing much beyond early life.^49^ Meanwhile, damping ratio-based models show a progressive decline in cortical relevance in older ages, revealing that cortical sensitivity to ageing—previously observed only during the developmental years (ages 5–35)^49^—does not persist into later adulthood. When considered individually, the caudate and thalamus emerge as the most age-sensitive subcortical structures. Previous MRE studies have primarily characterised the caudate and thalamus in terms of their age-related decline in stiffness, with an accelerated decrease observed in elderly individuals.^61^ Our voxel-wise approach extends these findings by not only confirming their role in age-related stiffness changes but also demonstrating their relevance for damping ratio-based biomarkers. In addition to previous findings on caudate atrophy and metabolic decline in ageing and neurodegeneration^62^^,^^63^, our results emphasise the importance of biomechanical characterisation in detecting these processes. Studies have shown that caudate atrophy is associated with gait dysfunction and poorer physical performance,^63^ while metabolic reductions in the caudate nucleus serve as sensitive biomarkers for normal ageing and early neurodegenerative stages.^62^ Furthermore, recent research highlights dopaminergic deficits in the caudate as a contributing factor to cognitive decline in Parkinson's disease, particularly in pre-dementia stages.^64^ Our findings extend this understanding by demonstrating that biomechanical measures offer a distinct and sensitive marker of age-related changes in the caudate, reinforcing its role as a key region in detection of ongoing neurodegeneration. Similarly, beyond well-documented atrophy and morphological alterations of the thalamus in neurodegenerative diseases^65^^,^^66^, our study highlights the potential of biomechanical properties as complementary biomarkers of ageing and disease progression. Thalamic morphology has been proposed as a putative biomarker across multiple neurodegenerative disorders^65^, with distinct patterns of atrophy observed in early- and late-onset Alzheimer's disease.^66^ Our results build on this by showing that stiffness and damping ratio capture differential ageing effects within thalamic subregions, further underscoring the relevance of MRE-derived biomarkers in tracking the progression of neurodegeneration.

Beyond healthy ageing, our contrastive regression framework enables the detection of neurodegeneration-related changes from MRE data. While MRI-derived models show minimal BAG differences, MRE reveals significant deviations. Our results reveal that damping ratio is more sensitive to early MCI-related neurodegeneration, while stiffness more effectively captures higher predicted brain age in AD. Damping ratio is often considered to reflect the microstructural organisation of tissue,^9^ and may capture subtle changes in tissue such as inflammation. This is consistent with prior findings that damping ratio is strongly associated with cognitive function and memory performance, that may be affected by similar changes in tissue microstructure,^67^^,^^68^ making damping ratio a potentially sensitive marker of early cognitive decline.^69^^,^^70^ In contrast, stiffness decline reflects advanced neurodegeneration, with significant reductions observed in patients with AD.^59^^,^^71^ Stiffness is affected by changes in tissue composition^9^ and the progressive loss of tissue structure from neuronal death is likely captured by this property. These findings further support the interpretation that mechanical properties reflect different aspects of neurodegeneration — with damping ratio changes more prominent in earlier stages (MCI) and stiffness reductions more pronounced in later stages (AD), although longitudinal data will be required to confirm temporal ordering. A noteworthy result is the large stiffness-based BAG observed in the thalamus of the AD cohort. This aligns with literature identifying the thalamus as a critical hub for AD pathology, with evidence that its degeneration can be an early event that contributes directly to cognitive symptoms.^72^^,^^73^ However, the relatively small sample size warrants caution and future larger studies are needed to confirm the magnitude and specificity of this effect.

Despite these advantages, several limitations remain, offering avenues for future improvement. Although our dataset is relatively large for MRE studies, it is still smaller than typical MRI-based datasets,^52^ which may partly explain the lack of statistical significance in MRI-based models for AD detection. Accordingly, we refrain from drawing firm conclusions from this observation. Similarly, while our self-supervised model has outperformed the baseline models in this study, a direct comparison with publicly available MRI-based brain age prediction methods lies beyond the scope of this work, as our primary objective has been to evaluate MRI and MRE under matched conditions. The higher MRI MAEs being observed here compared with those reported in large-scale MRI-only studies are consistent with our modest sample size. As highlighted by a recent study,^74^ model performance depends on multiple factors, including training dataset size and composition as well as preprocessing pipelines. Importantly, the ability to detect significant effects in a relatively small MRE sample supports the sensitivity of our approach, suggesting that MRE captures relevant biomechanical changes even under limited sample sizes using a self-supervised contrastive regression framework. Nonetheless, the modest size of our clinical cohorts is a limitation inherent to this emerging modality, and the clinical findings should therefore be interpreted with caution. Future large-scale studies will be essential to validate the promising effects observed here.

The inclusion of multiple clinical studies introduces potential confounders, which we have attempted to mitigate through image registration; however, residual inter-study variability may persist. Another limitation is the need to retrain models without healthy controls when computing brain age gaps for disease cohorts. While this approach reduces site-related confounding, it limits model generalisability, a challenge that larger and more diverse datasets will help to overcome. Current MRE studies also occasionally exhibit partial brain coverage, potentially affecting regional analyses. Brainstem measurements remain particularly challenging due to shear-wave attenuation in central brain regions and susceptibility to motion artefacts from adjacent pulsating structures. Continued advances in MRE hardware and sequence design will be crucial in addressing these issues.

Additional constraints arise from the retrospective nature of this study, which has precluded the inclusion of age-related comorbidities (e.g., vascular risk factors or metabolic conditions) known to affect brain structure and mechanics. Future prospective studies should incorporate these factors to better delineate their contribution. Finally, our occlusion analysis employed a 7 × 7 × 7 voxel mask, chosen to balance anatomical relevance with localisation in line with prior work. Systematic exploration of this parameter in future studies could further refine interpretability.

Our findings open promising avenues for future research. Within our framework, we have identified ‘healthy’ individuals who exhibit biomechanical brain-ageing patterns resembling those seen in patients with MCI or AD, despite scoring within the normal range on the Montreal Cognitive Assessment (MoCA). Whereas specialised neuropsychological composites such as the Preclinical Alzheimer's Cognitive Composite (PACC)^75^ are sensitive to subtle cognitive decline in preclinical AD, the MoCA is less sensitive than our image-derived brain-age biomarker. This raises the hypothesis that such biomechanical patterns reflect increased vulnerability to future cognitive decline. Alternatively, they may represent benign variants of normal ageing. Given the cross-sectional design of this study, we cannot distinguish between these possibilities, underscoring the need for longitudinal follow-up to determine whether these individuals later develop cognitive impairment or dementia. Moving forward, we plan to extend this framework to populations at risk for AD, identified through genetic predisposition and lifestyle factors, and to integrate MRE with multimodal approaches, including advanced MRI-based microstructural imaging,^76^ to improve sensitivity and strengthen clinical applicability.

In summary, our results establish that the contrastive regression framework offers a powerful and sensitive approach for detecting subtle, region-specific biomechanical changes linked to brain ageing and neurodegeneration, reflecting spatial heterogeneity across regions. By significantly enhancing the sensitivity of MRE-based models, this method paves the way for early, non-invasive detection of pathological ageing trajectories, even before clinical symptoms emerge. This represents a critical step toward presymptomatic screening and precision medicine in neurodegenerative diseases, with the potential to transform how we diagnose, monitor, and ultimately intervene in the ageing brain.

### Plain language summary

Our machine learning model is designed to estimate a person's brain age using a specialised type of scan called Magnetic Resonance Elastography (MRE). Unlike a standard MRI which shows the brain's structure, an MRE measures its biomechanical properties—specifically stiffness (how soft or firm the tissue is) and damping ratio (a property related to tissue viscosity). The goal is to use these properties to identify subtle, early signs of brain ageing and neurodegeneration. The overall workflow of our study, from data collection to clinical application, is illustrated in Fig. 1.

We trained our model on a dataset of 311 MRE scans from healthy individuals aged 14 to 90, collected across several research centres. To handle this moderately-sized dataset, we used an advanced AI technique called self-supervised contrastive learning. The model's performance was then tested on scans from patients with Alzheimer's disease (AD) and Mild Cognitive Impairment (MCI).

Our model can predict a person's age from their MRE scan with an average error of just 3.51 years, which was significantly more accurate than a model using standard MRI scans. The model learnt that changes in stiffness were a strong indicator of the more advanced changes seen in AD, while the damping ratio was more sensitive to the earlier changes in MCI. Our analysis also highlighted that deep brain structures, particularly the caudate and thalamus, were critical regions for tracking the effects of ageing.

This work is based on a retrospective analysis of existing, de-identified data. Our promising results suggest the potential for this MRE-based approach to serve as a non-invasive biomarker for brain health. Future longitudinal studies will be a crucial next step to validate these findings and to confirm if the biomechanical profiles we identified can help predict an individual's risk of cognitive decline. Such confirmation could pave the way for using this method to stratify at-risk individuals for timely inclusion in clinical intervention trials.

## Contributors

JT performed all the experiments and wrote the manuscript, LVH provided some of the study data and contributed to the writing of manuscript, CLH provided most of the study data and contributed to the writing of the manuscript, AVR helped with the supervision and contributed to the writing, CBS helped with the supervision, GSKS conceptualised the project, helped with the writing, provided the funding and supervised the project. JT and GSKS have accessed and verified the underlying data. All authors have read and approved the final version of the manuscript.

## Data sharing statement

Data from all studies can be accessed through a formal data-sharing agreement with CLJ via clj@udel.edu. Specifically, data from Study 1 and Study 2 are available at MRE134 and NITRC, respectively. There was no patient or public involvement in this study. The code is written in Python and PyTorch was used for model implementation. The code is publicly at https://github.com/CoderJacques/contrastive-MRE.

## Declaration of interests

All authors declare no competing interests.
