# Supplementary material for: Brain age prediction and early neurodegeneration detection using contrastive learning on brain biomechanics: a retrospective, multicentre study
Source: eBioMedicine. 2025 Oct 31;121:105996. doi: 10.1016/j.ebiom.2025.105996 (PMC12616099; doi:10.1016/j.ebiom.2025.105996)
Supplement: Supplementary Material [file mmc1.docx]

*Supplementary Material*

**Brain age prediction and early neurodegeneration detection using contrastive learning on brain biomechanics: a retrospective, multicentre study**

Jakob Träuble, MPhil^1^∙Lucy V Hiscox, PhD^2^∙Prof Curtis L Johnson, PhD^3^∙Prof Angelica Aviles-Rivero, PhD^4^∙Prof Carola B Schönlieb, PhD^5^∙Prof G Kaminski Schierle, PhD^1^

^1^Department of Chemical Engineering and Biotechnology, University of Cambridge, Cambridge, United Kingdom

^2^Cardiff University Brain Research Imaging Centre (CUBRIC), School of Psychology, Cardiff University, Cardiff, United Kingdom

^3^Department of Biomedical Engineering, University of Delaware, Newark, USA

^4^Yau Mathematical Sciences Center, Tsinghua University, China

^5^Department of Applied Mathematics and Theoretical Physics, University of Cambridge, Cambridge, United Kingdom

Emails & ORCIDs

Jakob Träuble, [jnt27@cam.ac.uk](mailto:jnt27@cam.ac.uk), ORCID: 0009-0007-2619-9395

Lucy Hiscox, [HiscoxL@cardiff.ac.uk](mailto:HiscoxL@cardiff.ac.uk), ORCID: 0000-0001-6296-7442

Curtis Johnson, [clj@udel.edu](mailto:clj@udel.edu), ORCID: 0000-0002-7760-131X

Angelica Aviles-Rivero, [aviles-rivero@tsinghua.edu.cn](mailto:aviles-rivero@tsinghua.edu.cn), ORCID: 0000-0002-8878-0325

Carola-Bibiane Schönlieb, [cbs31@cam.ac.uk](mailto:cbs31@cam.ac.uk), ORCID: 0000-0003-0099-6306

Gabriele Kaminski Schierle, [gsk20@cam.ac.uk](mailto:gsk20@cam.ac.uk), ORCID: 0000-0002-1843-2202

Correspondence to:

Prof Gabriele S Kaminski Schierle, PhD

Department of Chemical Engineering and Biotechnology

University of Cambridge

Cambridge

United Kingdom

gsk20@cam.ac.uk

**Supplementary - Materials & Methods**

**Data**

We compiled a dataset of 311 healthy individuals and 31 patients from multiple clinical studies. Each sample contains stiffness and damping ratio maps, as well as a T1-weighted anatomical scan. The healthy cohort was aggregated from five independent studies, each using highly similar MRE acquisition protocols and covering a broad age range (mean age: 41.0 ± 21.9 years). In addition, we assembled two disease cohorts comprising 31 patients, including 20 individuals classified with Mild-Cognitive Impairment^1^, and 11 individuals diagnosed with Alzheimer's Disease^2^. All imaging was performed on Siemens 3T MRI systems. Specifically, the five studies used either a Siemens 3T Prisma (University of Delaware) or Siemens 3T Trio (University of Illinois), while the patient cohorts were scanned on Siemens 3T Prisma (MCI cohort) and Siemens 3T Verio (AD cohort). Thus, while different Siemens 3T models were used across sites, all data were acquired at 3T field strength from the same vendor. All studies used a Resoundant pneumatic actuator pillow system with a frequency of 50 Hz and a 3D spiral MRE sequence^3,4^, and were anonymised prior to analysis. Sex was self-reported by participants at study intake. This dataset represents one of the largest collections of MRE-derived brain mechanical properties to date. Detailed information of our dataset can be found in [Supplementary Table 1](#tab1).

**Supplementary Table 1: Detailed dataset information.** *: This study is a pooled study itself.

|  | **Study** | **Published In** | **#Subjects** | **Age [Years]** | **Sex [F:M]** | **Resolution [mm^3^]** |
| --- | --- | --- | --- | --- | --- | --- |
| Healthy | **1*** | ^5^ | 134 | 23.4 ± 4.0 | 78:56 | 2.0 |
|  | 2 | ^6^ | 60 | 37.8 ± 20.9 | 34:26 | 1.5 |
|  | 3 | ^2^ | 12 | 69.4 ± 2.4 | 6:6 | 1.6 |
|  | 4 | ^1^ | 68 | 69.3 ± 5.8 | 49:19 | 1.25 |
|  | 5 | ^7,8^ | 37 | 49.1 ± 16.6 | 16:21 | 1.25 |
|  | **Total** | **-** | **311** | **41.0 ± 21.9** | **183:128** | **2.0** |
| Disease | 6 | ^2^ | 11 | 76.8 ± 5.3 | 7:4 | 1.6 |
|  | 7 | ^1^ | 20 | 72.6 ± 8.5 | 14:6 | 1.25 |

Understanding the age distribution of our dataset is essential for evaluating the generalizability of brain age prediction models. [Supplementary Fig. S1](#FigS1_) presents the combined age distribution from all pooled studies contributing to the MRE dataset, with each study's contribution highlighted in different colours. The distribution exhibits a bi-modal pattern, characterised by two predominant age clusters corresponding to younger and older cohorts. This pattern arises due to the inherent limitation of a lack of middle-aged volunteers in clinical trials. To mitigate this imbalance, we employ the adaptive neighbourhood approach, which compensates for dataset non-uniformity through the contrastive regression loss function. This method ensures that age predictions remain robust despite the dataset’s skewed age distribution, enhancing the model's ability to generalise across age groups.

**Supplementary Figure 1: Age Distribution of Pooled Studies of MRE Dataset.** Contribution of each study is highlighted in colour. The distribution shows bi-modal characteristics with two predominant age groups among samples.

**Image Pre-Processing**

Displacement fields obtained from MRE acquisitions were processed using the nonlinear inversion (NLI) algorithm^9^, which estimates the complex shear modulus $G* = G' + iG''$. Here, G' represents the storage modulus, while G'' corresponds to the loss modulus. From these values, we derived key mechanical properties: the stiffness measure μ as $\frac{2|{G*|}^{2}}{G' + |G*|}$ and the damping ratio ξ as $\frac{G''}{2G'}$ ^10,11^. These computed maps of stiffness and damping ratio were used as inputs for further analysis. Next, the MRE magnitude map and T1-weighted scans underwent skull stripping using FreeSurfer^12^ to extract brain tissue while removing non-brain structures. A bias field correction was then applied to eliminate intensity gradients that could introduce inconsistencies in the analyses. To mitigate inter-study variability, we performed affine registration of the images to the MNI152 template. We chose this approach as it is a standard and widely adopted method for providing anatomical correspondence in a common space for voxel-wise deep learning analyses of brain MRI^13,14^. This registration was carried out using ANTs^15^ at an isotropic resolution of 2 mm³, with final image dimensions of 91 × 109 × 91, ensuring uniform orientation and scale across all data. Finally, we normalised the quantitative stiffness and damping ratio maps by Z-scoring, adjusting their mean to zero and standard deviation to one across the entire dataset. This normalisation approach preserves global trends of brain softening and increasing viscoelasticity with age and was consistently applied throughout the analysis. An exception was made for the analysis in [Supplementary Fig. S2](#FigS2_), where each image was independently normalised to a zero mean and unit standard deviation to specifically examine the spatial distribution of mechanical properties across ageing. T1-weighted images were Z-scored on a per-image basis using the same standardisation method. Furthermore, to account for variability in brain coverage within the quantitative mechanical property maps, T1-weighted images were masked accordingly to ensure consistent coverage across modalities.

**Contrastive Regression Framework for Brain Age Estimation**

Typically, **self-supervised and contrastive learning approaches** are primarily designed for **classification tasks**, where samples are grouped into discrete categories. However, brain age prediction is a **regression problem**, requiring a fundamentally different formulation where similarity is determined by **continuous age differences** rather than class labels. Thus, we employ a **contrastive regression framework** designed to predict brain age from **MRE-derived stiffness and damping ratio maps, as well as MRI images**. This approach extends the previously developed **Localised Neighbourhoods method**^16^**,** which employs contrastive learning to adaptively adjust sample neighbourhoods in non-uniform regression tasks.

Unlike classification-based contrastive learning, where positive and negative pairs are predefined, our **contrastive regression loss** ensures that samples are attracted or repelled based on their **age similarity**:

$$L_{\text{contrastive}}=-\sum_{i} \sum_{k\neq i} w_{i,k}\log\frac{\exp\left( s_{i,k} \right)}{\sum_{t\neq k} \exp\left( s_{i,t} \right)}$$

where:

- s_i,k_ is the **cosine similarity** between embeddings 𝑓(x_i_) and 𝑓(x_k_).
- w_i,k_is an **age-aware weighting function**, defining sample similarity based on age difference.

Following Barbano et al.^17^, we integrate:

1. **Y-Aware weighting**, where w_i,k_ =K(y_i_−y_k_) controls sample attraction/repulsion strength using a **Gaussian kernel**:

$$K\left( y_{i}-y_{k} \right)=\exp\left( -\frac{\left( y_{i}-y_{k} \right)^{2}}{2\sigma^{2}} \right)$$

This ensures that **age-similar samples** are drawn closer while distant ones are repelled.

1. **Exponential Scaling,** modifying the denominator to increase repulsion strength for dissimilar samples:

$$L_{\text{exp}}=-\sum_{i} \sum_{k\neq i} w_{i,k}\log\frac{\exp\left( s_{i,k} \right)}{\sum_{t\neq k} \exp\left( s_{i,t}\left( 1-w_{i,t} \right) \right)}$$

which adjust repulsion strength based on the samples’ age difference.

Further, following the Adaptive Neighbourhoods’ approach^16^, our method **progressively refines**sample neighbourhoods during training:

$$\mathcal{NN}\left( x_{i}; \text{epoch} \right)= \{ x_{k} \mid f_{\text{epoch}}\left( x_{k} \right) \text{ is among the } \mathcal{N}\mathcal{N}_{\text{n}\text{b}}\left( \text{epoch} \right) \text{ nearest neighbo}\text{u}\text{rs of }f_{\text{epoch}}\left( x_{i} \right) \text{ based on }d\left( f_{\text{epoch}}\left( x_{k} \right),f_{\text{epoch}}\left( x_{i} \right) \right) \}$$

where:

- NN_nb_ is the number of nearest neighbours, which is progressively reduced during training,
- d(𝑓(x_i_), 𝑓(x_k_)) is the distance between the embeddings 𝑓(x_i_) and 𝑓(x_k_).

This allows early learning to capture **broad ageing patterns**, while later training**refines local age-related differences**, expressed in the adaptive neighbourhood contrastive regression loss:

$$L_{\text{AdapNN}}=-\sum_{i} \sum_{k\neq i} \frac{w_{i,k}}{\sum_{t} w_{i,t}}\log\left( \frac{\exp\left( s_{i,k} \right)}{\sum_{x_{t}\in\text{NN}\left( x_{i};\text{epoch} \right)} \exp\left( s_{i,t}\left( 1-w_{i,t} \right) \right)} \right)$$

**Evaluation Protocol**

Our evaluation strategy comprises three distinct modelling approaches: a kernel-based method, an end-to-end supervised CNN, and a self-supervised method. The first two approaches, kernel-based methods and supervised deep learning, are widely used in brain age prediction studies. Specifically, we apply principal component analysis (PCA) for feature extraction, followed by Gaussian process regression (GP), as a representative kernel method. Additionally, we implement a supervised deep learning model using a ResNet^18^ convolutional neural network (CNN) to learn end-to-end age-relevant features directly from the input data. We selected ResNet-18 as the supervised baseline due to its balance between model capacity and dataset scale. From a statistical learning theory perspective, deeper or more complex architectures would risk overfitting given our sample size and 3D input dimensionality, while simpler architectures could underfit^19^. ResNet-18 has been shown to converge reliably in volumetric medical imaging studies^20^ and provides a fair comparison across modalities under consistent architecture and training protocols. Beyond these established approaches, we employ the proposed self-supervised learning framework, which uses adaptive neighbourhood sampling to learn meaningful representations for regression tasks. All models were trained using an 80:20 train-test split. Model performance was assessed using the mean absolute error (MAE) on the test set, averaging the results across ten random seeds.

For the kernel-based approach, we employ PCA for feature selection, followed by GPs with linear kernels. The models were trained for 50 epochs using the Adam optimiser, with Gaussian noise serving as an augmentation technique for regularisation. Hyperparameter optimisation was conducted via random search across 50 iterations for each modality: stiffness-only, damping ratio-only, MRE (damping ratio + stiffness, concatenated and input into PCA), and MRI. The hyperparameters tuned included: Learning rate (lr): [0.0001, 0.001, 0.01, 0.1], PCA components (pca k): [10, 50, 100], Noise standard deviation (std): [0.02, 0.05, 0.1, 0.15, 0.2]. The optimal hyperparameter configurations selected were:

- **Stiffness-only**: lr = 0.001, PCA k = 100, noise std = 0.02
- **Damping ratio-only**: lr = 0.001, PCA k = 100, noise std = 0.1
- **MRE**: lr = 0.1, PCA k = 100, noise std = 0.15
- **MRI**: lr = 0.1, PCA k = 100, noise std = 0.2

For the end-to-end supervised CNN, we selected ResNet-18, the smallest ResNet variant, to align with the dataset scale. The architecture consists of channel dimensions: [input channel, 16, 32, 64, 128]; input channels: 1 for MRI, 2 for MRE; latent head with an embedding dimension of 256; a regression head consisting of a two-layer MLP (256🡪64🡪1); ReLU activation throughout the network; using Kaiming initialisation. Models were trained for 50 epochs using the L1 loss function, a batch size of 32, a learning rate of $5 \times{10}^{-5}$ and the Adam optimiser. Regularisation was implemented using Gaussian noise augmentation, weight decay, and dropout. Hyperparameter tuning was performed via random search across 20 iterations, optimizing: Dropout rate (dropout): [0.1, 0.2], Weight decay: [0.00005, 0.0001], Noise standard deviation (std): [0.02, 0.05, 0.1, 0.15, 0.2]. The optimal hyperparameter configurations selected were:

- **Stiffness-only**: dropout = 0.1, weight decay = 0.0001, noise std = 0.2
- **Damping ratio-only**: dropout = 0.1, weight decay = 0.0001, noise std = 0.1
- **MRE**: dropout = 0.1, weight decay = 0.00005, noise std = 0.15
- **MRI**: dropout = 0.1, weight decay = 0.00005, noise std = 0.15

For the self-supervised approach, the same ResNet-18 architecture is trained in a self-supervised manner using the Adaptive Neighbourhood Contrastive Regression Loss. Following the training of the representations, we employed a Ridge Regression estimator on top of the frozen encoder to predict age. Models were trained for 50 epochs using the Adam optimiser, a batch size of 32, an initial learning rate of $1 \times{10}^{-4}$, and a stepwise learning rate decay (reduced by 0.9 every 10 epochs) to calculate the degrees of positiveness of pairs. Hyperparameter tuning, performed via random search across 10 iterations, included: Distance metric: [Manhattan, Euclidean, Similarity], Weight decay: [0.00005, 0.0001], Noise standard deviation (std): [0.1, 0.15, 0.2], Nearest neighbour step size: [1, 2, 5], End nearest neighbour count: [8, 9, 10, 11, 12, 13, 14]. The optimal hyperparameter configurations selected were:

- **Stiffness-only**: distance = Euclidean, weight decay = 0.00005, noise std = 0.15, NN step size = 2, end NN count = 8
- **Damping ratio-only**: distance = Euclidean, weight decay = 0.0001, noise std = 0.2, NN step size = 1, end NN count = 14
- **MRE**: distance = Manhattan, weight decay = 0.00005, noise std = 0.1, NN step size = 5, end NN count = 9
- **MRI**: distance = Euclidean, weight decay = 0.00005, noise std = 0.15, NN step size = 1, end NN count = 12

To facilitate the analysis of the impact of spatial normalisation (Supplementary Fig. S2), we repeated the same evaluation protocol and hyperparameter tuning using the alternative normalisation strategy where images are normalised to mean zero and standard deviation of one on image-level rather than dataset-level. This adjustment allows us to investigate spatial effects of ageing independent of global large-scale softening and viscoelasticity trends. The same hyperparameter tuning settings were used as in the primary evaluation. The optimal hyperparameter configurations for GPs were: stiffness-only (lr = 0.01, pca k = 100, noise std = 0.2) and damping ratio-only (lr = 0.001, pca k = 100, noise std = 0.2. For the ResNet-based supervised learning approach, the best configurations were: stiffness-only (dropout = 0.1, weight decay = 0.0001, noise std = 0.02) and damping ratio-only (dropout = 0.1, weight decay = 0.00005, noise std = 0.2). In the self-supervised setting, the optimal model selections were: stiffness-only (distance = Euclidean, weight decay = 0.00005, noise std = 0.1, NN step size = 2, end NN count = 8) and damping ratio-only (distance = Euclidean, weight decay = 0.00005, noise std = 0.15, NN step size = 1, end NN count = 13).

**Saliency Maps**

To investigate the spatial importance of brain regions for age prediction, we conducted an occlusion sensitivity analysis^21^ using the trained self-supervised model, the test dataset and the dataset-wide normalisation. This approach allows us to track how mechanical properties influence brain age predictions across different life stages. Test samples were divided into five sub-age groups based on chronological age quantiles. For each group, we systematically occluded 7×7×7 voxel regions by replacing them with zero values. To reduce computational overhead, the superior-most 9 slices along the superior-inferior axis —corresponding to non-brain areas — were excluded from occlusion. After occlusion, the model was used to generate age predictions, and the effect of occlusion was quantified as the difference in mean absolute error (MAE) between occluded and original predictions (delta MAE). Iterating this process for all occlusion regions resulted in a delta MAE matrix of size 13×13×13 (n = 2,197). To focus on relevant variations, values were clipped between the 5th and 95th percentiles. The delta MAE matrix was then resized to match the original image dimensions (91×109×91) using cubic interpolation, with zero padding applied to the excluded areas. Finally, the reconstructed saliency map was normalised by min-max scaling values between 0 and 1.

**Brain Age Gaps**

To assess brain age gaps (BAGs) in disease cohorts, models were retrained (with modality-specific best hyperparameters identified in the whole-brain analysis) using only healthy samples while excluding the healthy control subset from the applicable disease cohort. These models were then used to predict ages for both the disease and control cohorts. To correct for systematic biases, a Theil-Sen Regressor was fitted on the control cohort and subsequently applied to the disease cohort. The Brain Age Gap was then computed as the difference between the corrected predicted age and the chronological age for each individual.

**Regional Analysis**

To evaluate the regional contributions of brain structures to brain age prediction, brain region-specific masks were applied, and separate models were trained for each region using the best hyperparameters for the specific modality identified in the whole-brain analysis. These models were trained exclusively on healthy samples to establish baseline ageing trajectories for different brain regions. For the regional BAG analysis, the same workflow as the whole-brain BAG analysis was followed, where the healthy control cohort was excluded from the training set. The trained region-specific models were subsequently applied to both disease and control cohorts, and the Theil-Sen correction was fitted on the control cohort samples. Individual brain age profiles were computed for each participant and clipped between the 5th and 95th percentiles to ensure robustness against outliers. To facilitate cohort-level interpretations, individual profiles were averaged within each disease group to generate representative cohort-specific brain age profiles.

**Supplementary - Results**

**Supplementary Figure 2: Predicted vs. chronological age across modalities.** Scatter plots on the held-out test set for one representative run per modality: T1-weighted MRI, damping ratio, stiffness and combined MRE (stiffness + damping ratio). The solid line indicates identity. Per-panel MAE and R^2^ are annotated.

**Localised ageing effects in spatially normalised mechanical properties confirm MRE’s sensitivity beyond global trends**

**Supplementary Figure 3:** **Disentangling global and local ageing effects: Impact of spatial normalisation on MRE-based predictions.** Evaluating spatially normalised mechanical properties highlights the predictive value of localised ageing effect. Stiffness proves to be the stronger predictor in all spatially normalised models.

To better understand the effects of global versus local ageing patterns, we evaluated brain age prediction using spatially normalised MRE scans (see [Supplementary Fig. S3](#FigS2_)). Unlike our primary analysis, which retained global trends in stiffness and damping ratio, this approach normalises each scan to zero mean and unit variance before training. By removing large-scale mechanical ageing trends, this normalisation isolates spatially localised ageing effects. Under the spatially normalised conditions, stiffness outperforms damping ratio for kernel-based methods. PCA+GPs achieve an MAE of 7.12 years for stiffness, a 5.1% improvement over damping ratio (MAE = 7.50 years). Similarly, in deep learning models, stiffness emerges as the stronger predictor. Supervised learning reduces the MAE to 6.36 years for stiffness compared to 7.67 years for damping ratio, while self-supervised learning further improves stiffness-based predictions to an MAE of 4.22 years. Compared to the previous normalisation method, which preserved global ageing trends, supervised and self-supervised deep learning models show slightly higher MAEs, highlighting the predictive value of large-scale mechanical changes. However, this analysis shows that spatially localised distribution changes contain key information for brain age prediction.

**Combining MRI and MRE data improves brain age prediction compared to MRI alone**

**Supplementary Figure 4:** **Brain age prediction when combining MRI with MRE brain maps.** Mean absolute error (MAE) of age prediction for the three different modelling approaches: PCA with Gaussian Processes, supervised deep learning, and self-supervised deep learning.

To evaluate the added value of combining imaging modalities for brain age prediction, we compared the performance of models using both MRI and MRE images (see [Supplementary Figure 4](#FigS4)). Models incorporating MRE data on top of MRI data outperform those trained on MRI data alone. When comparing the combined MRI+MRE models to the MRE-only models, we observed similar performance levels. For the PCA-based and supervised deep learning models, the MRE-only approach yielded slightly lower mean absolute errors (MAEs) of 6.51 and 4.69 years, respectively, compared to the combined models' MAEs of 6.54 and 5.09 years. For the self-supervised deep learning approach, the combined model achieved the lowest MAE of 3.42 years, showing a small improvement over the MRE-only model (3.51 years). These results highlight that MRE captures much of the age-relevant information on its own, while MRI adds complementary features that can further refine prediction performance in certain modelling frameworks. In particular, the self-supervised approach appears best suited to integrate the two modalities, suggesting that representation learning can exploit subtle synergies between MRI and MRE. Overall, this indicates that the benefit of multimodal integration depends strongly on the modelling strategy.

**References**

1 Delgorio PL, Hiscox LV, McIlvain G, *et al.* Hippocampal subfield viscoelasticity in amnestic mild cognitive impairment evaluated with MR elastography. *NeuroImage Clin* 2023; **37**: 103327.

2 Hiscox LV, Johnson CL, McGarry MDJ, *et al.* Mechanical property alterations across the cerebral cortex due to Alzheimer’s disease. *Brain Commun* 2020; **2**: fcz049.

3 Johnson CL, Holtrop JL, McGarry MDJ, *et al.* 3D multislab, multishot acquisition for fast, whole‐brain MR elastography with high signal‐to‐noise efficiency. *Magn Reson Med* 2014; **71**: 477–85.

4 McIlvain G, Cerjanic AM, Christodoulou AG, McGarry MDJ, Johnson CL. OSCILLATE : A low‐rank approach for accelerated magnetic resonance elastography. *Magn Reson Med* 2022; **88**: 1659–72.

5 Hiscox LV, McGarry MDJ, Schwarb H, *et al.* Standard‐space atlas of the viscoelastic properties of the human brain. *Hum Brain Mapp* 2020; **41**: 5282–300.

6 Bayly PV, Alshareef A, Knutsen AK, *et al.* MR Imaging of Human Brain Mechanics In Vivo: New Measurements to Facilitate the Development of Computational Models of Brain Injury. *Ann Biomed Eng* 2021; **49**: 2677–92.

7 Sanjana F, Delgorio PL, Hiscox LV, *et al.* Blood lipid markers are associated with hippocampal viscoelastic properties and memory in humans. *J Cereb Blood Flow Metab* 2021; **41**: 1417–27.

8 Delgorio PL, Hiscox LV, Daugherty AM, *et al.* Structure–Function Dissociations of Human Hippocampal Subfield Stiffness and Memory Performance. *J Neurosci* 2022; **42**: 7957–68.

9 McGarry MDJ, Van Houten EEW, Johnson CL, *et al.* Multiresolution MR elastography using nonlinear inversion. *Med Phys* 2012; **39**: 6388–96.

10 Manduca A, Oliphant TE, Dresner MA, *et al.* Magnetic resonance elastography: Non-invasive mapping of tissue elasticity. *Med Image Anal* 2001; **5**: 237–54.

11 McGarry MDJ, Van Houten EEW. Use of a Rayleigh damping model in elastography. *Med Biol Eng Comput* 2008; **46**: 759–66.

12 Fischl B. FreeSurfer. *NeuroImage* 2012; **62**: 774–81.

13 Cole JH, Poudel RPK, Tsagkrasoulis D, *et al.* Predicting brain age with deep learning from raw imaging data results in a reliable and heritable biomarker. *NeuroImage* 2017; **163**: 115–24.

14 Mouches P, Wilms M, Rajashekar D, Langner S, Forkert ND. Multimodal biological brain age prediction using magnetic resonance imaging and angiography with the identification of predictive regions. *Hum Brain Mapp* 2022; **43**: 2554–66.

15 Gang Song BBA. Advanced normalization tools (ants). *Insight J* 2009; **2(365)**: 1–35.

16 Träuble J, Hiscox L, Johnson C, Schönlieb C-B, Schierle GK, Aviles-Rivero A. Contrastive Learning with Adaptive Neighborhoods for Brain Age Prediction on 3D Stiffness Maps. *Trans Mach Learn Res* 2024; published online Nov.

17 Barbano CA, Dufumier B, Duchesnay E, Grangetto M, Gori P. Contrastive Learning for Regression in Multi-Site Brain Age Prediction. In: 2023 IEEE 20th International Symposium on Biomedical Imaging (ISBI). Cartagena, Colombia: IEEE, 2023: 1–4.

18 He K, Zhang X, Ren S, Sun J. Deep residual learning for image recognition. 2016: 770–8.

19 Vapnik VN, Vapnik VN. Statistical learning theory. New York Weinheim: Wiley, 1998.

20 Kamnitsas K, Ledig C, Newcombe VFJ, *et al.* Efficient multi-scale 3D CNN with fully connected CRF for accurate brain lesion segmentation. *Med Image Anal* 2017; **36**: 61–78.

21 Lee J, Burkett BJ, Min H-K, *et al.* Deep learning-based brain age prediction in normal aging and dementia. *Nat Aging* 2022; **2**: 412–24.
